# Pain Incidence and Associated Risk Factors among Cancer Patients within 72 Hours after Surgery: A Large Retrospective Analysis

**DOI:** 10.3390/curroncol30010065

**Published:** 2023-01-08

**Authors:** Junlan Qiu, Yirong Xin, Jiazhen Yao, Lingkai Xu, Fang Meng, Lin Feng, Xiaochen Shu, Zhixiang Zhuang

**Affiliations:** 1 Department of Oncology, The Second Affiliated Hospital of Soochow University, Suzhou 215004, China; 2 Department of Oncology and Hematology, The Suzhou Science & Technology Town Hospital, Suzhou 215153, China; 3 Department of Epidemiology, School of Public Health, Soochow University, Suzhou 215123, China; 4 Department for Communicable Disease Control and Prevention, Suzhou Wuzhong Center for Disease Prevention and Control, Suzhou 215128, China; 5 Institute of Systems Medicine, Chinese Academy of Medical Sciences & Peking Union Medical College, Beijing 100005, China; 6 Suzhou Institute of Systems Medicine, Suzhou 215123, China; 7 Jiangsu Key Laboratory of Preventive and Translational Medicine for Geriatric Diseases, Medical College of Soochow University, Suzhou 215123, China

**Keywords:** cancer patients, postoperative pain, pain management, adverse reactions, risk factors

## Abstract

Background: A fundamental principle of pain management is to determine the distribution and causes of pain. However, relevant data among postoperative cancer patients based on a large amount of data remain sparse. Objective: We aimed to investigate the incidence of postoperative pain in cancer patients and to explore the associated risk factors. Methods: We retrospectively collected information on postoperative pain-evaluation records of cancer patients who underwent surgery between 1 January 2014 and 31 December 2019. Descriptive statistics were presented, and multinominal logistic regression analysis was performed to explore the risk factors associated with postoperative pain. Results: Among the 11,383 patients included in the study, the incidence of mild/moderate to severe pain at the 24th hour after surgery was 74.9% and 18.3%, respectively. At the 48th and 72nd hour after surgery, the incidence of mild pain increased slightly, while the incidence of moderate to severe pain continued to decrease. Female patients experienced a higher risk of pain (ORs: 1.37–1.58). Undergoing endoscopic surgery was associated with a higher risk of pain (ORs: 1.40–1.56). Patients with surgical sites located in the respiratory system had a higher risk of pain compared to in the digestive system (ORs: 1.35–2.13), and other patients had a relatively lower risk of pain (ORs: 0.11–0.61). Conclusion: The majority of cancer patients experienced varying degrees of postoperative pain but may not receive adequate attention and timely treatment. Female, young age and endoscopic surgery were associated with increased pain risk, and effective identification of these high-risk groups had positive implications for enhanced postoperative pain management.

## 1. Introduction

Pain is one of the most prevalent and distressing symptoms for cancer patients. In 1985, Bonica evaluated the prevalence of cancer pain (CP) in 15 countries worldwide and found that the average prevalence of pain for all stages of cancer was 50% [1]. The World Health Organization (WHO) established guidelines for the management of cancer pain in 1986, and these guidelines have subsequently been used more widely throughout the world [2], but there are numerous studies suggesting that the treatment status of cancer pain remains unsatisfactory [3,4,5]. Related studies have shown that of the 2.8 million Chinese patients with advanced cancer, 80% experienced pain and 70% did not receive effective treatment [6]. Pain as a variable affecting health-related quality of life (HRQOL) is of great clinical importance [7,8]. Poor pain control is associated with more psychological distress and decreased social activities and social support [9]. In addition, pain can also lead to reduced patient compliance with antineoplastic treatment, which is detrimental to the treatment of and recovery from the disease [10]. Given the prevalence and adverse effects of pain, scientific assessment of pain and effective pain management are critical.

Because cancer pain is not a directly measurable entity, proper pain assessment is a prerequisite and foundational for effective pain management. Cancer pain is a widely used term for a range of different pain conditions, and the complexity of its etiology and pathogenesis is often emphasized [11,12]. Therefore, identifying demographic and clinical factors that may be associated with pain after surgical operation is important, and is of great significance for future postoperative pain control and management. Although there have been some studies on the incidence and risk factors of postoperative pain in patients to date, most of them have a relatively small numbers of subjects; for example, in a retrospective study of moderate to severe postoperative pain in patients undergoing televised thoracoscopy, a total of 1164 patients were included for correlation analysis [13], or the number of participants was adequate but the study focused only on the first 24 h after surgery [14]. Studies describing the pain experience and associated risk factors of cancer patients within 72 h after surgery based on a large amount of data are scarce.

In the present study, our goal was to investigate the incidence of postoperative pain in 11,383 cancer patients and to explore risk factors associated with postoperative pain. Our study had a larger study population and extended the observation period to the 72nd hour postoperatively for patients. In addition, our study also involved information on trends in patients’ pain intensity and the incidence of adverse reactions over time postoperatively. The results could help clinicians perform more scientific and effective pain management, thus reducing patients’ pain and improving their postoperative quality of life.

## 2. Materials and Methods

### 2.1. Study Design and Population

**Figure 1:**
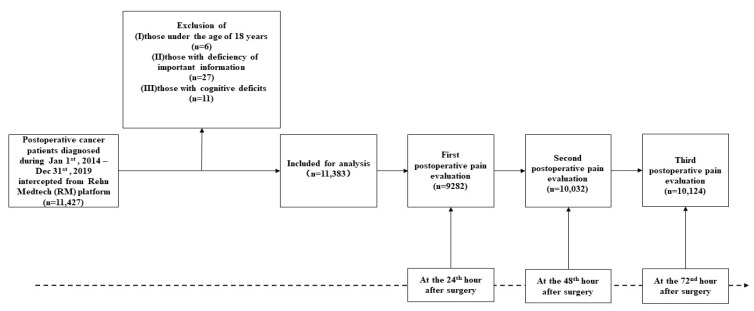
Flow diagram of study participants.

This retrospective, observational study was conducted in Nantong Tumor Hospital in China. A total of 11,427 inpatients were recruited according to the inclusion criteria, as follows: (1) patients with tumors; (2) received their first surgical treatment for tumor at Nantong Cancer Hospital between 1 January 2014 and 31 December 2019; and (3) hospitalized for observation for more than three days after surgery. As shown in Figure 1, after excluding ineligible patients according to the exclusion criteria, the study population ultimately included 11,383 patients. Cases of multiple surgeries were identified and excluded from the analysis in the present study considering its violation of one of the core assumptions of the regression modeling, which is that the events/observations need to be observed at random and they also need to be independent. The specific exclusion criteria were as follows: (1) under the age of 18 years; (2) severely missing basic information; and (3) have cognitive deficits. 

### 2.2. Data Source and Collection

All data used in the study were extracted from the health data platform of Rehn Medtech (RM: www.rehn.cc, accessed on 1 July 2021) (Jiangsu, China), which is a big-data intelligence platform that integrates and converges mass (over 100 hospitals) multi-source heterogeneous electronic health-record data from multiple medical centers or hospitals all over China. Data were initially extracted by the platform’s professionally qualified data engineers using a dedicated server and exported from the data system for further collation and analysis by researchers. The data system could set a reasonable range of values for variables, and abnormal data could be automatically identified to avoid errors in data extraction and transmission as much as possible and to ensure data integrity and reliability. The initial extracted data were further collated and analyzed by two researchers independently, and the data processing process and results were reproducible. Missing data were marked and scientifically processed according to the actual situation of the study, and duplicate or invalid data were effectively identified and reasonably handled at an early stage of data collation. Information extracted for our study from this platform’s electronic health records, included the patient’s gender, age, weight, surgical modality, surgical site, type of medication used for analgesia and NRS pain scores after surgery.

### 2.3. Pain Intensity Assessment as Outcome

Proper assessment of pain intensity is an important prerequisite for pain description and pain management. Intensity, as an important characteristic of pain, is considered the gold standard for performing pain assessment. Therefore, intensity is often used clinically as a pain indicator to perform pain-level assessment and to guide the choice of treatment plan [15]. There are various methods of measuring intensity, of which we adopted the most-used one, numerical rating scale (NRS), in the present study. The NRS pain scale divides pain into 11 levels, and patients can assess pain levels by choosing from a scale of 0–10 (0 for no pain at all and 10 for the most intense pain imaginable) depending on their pain experience. Using this method, three pain evaluations were performed at the 24th, 48th and 72nd hour after the patient’s surgery. Patients were divided into four groups based on NRS scores: no pain (NRS = 0), mild pain (NRS = 1–3), moderate pain (NRS = 4–6) and severe pain (NRS = 7–10). Due to the limited number of patients with severe pain, moderate pain (NRS = 4–6) and severe pain (NRS = 7–10) were thus combined for analysis, i.e., moderate to severe pain (NRS = 4–10). In addition to the pain assessment, we also conducted an investigation of the occurrence of adverse effects, using a pre-designed questionnaire in the evaluation system which included common postoperative adverse reactions. Patients chose the relevant symptoms according to their conditions during the postoperative adverse reaction assessment, with the help of medical staff.

### 2.4. Statistical Analysis

In our study, frequency distributions were calculated and descriptive statistics were performed based on information such as demographics and clinical characteristics. A total of six variables were included in the analysis: gender; age; weight; surgical modality; system in which the surgical site was located; and type of medication used. The surgical modality was divided into two types: endoscopic surgery and non-endoscopic surgery. The surgical sites were classified according to the system to which the organ in which they were located belonged. For example, sites such as lung and trachea were classified as respiratory system; sites such as gastrointestinal tract, liver, and gallbladder were classified as digestive system; sites such as kidney and bladder were classified as urinary system; etc. [16]. In total, surgical sites were classified into six categories: digestive; respiratory; reproductive; urinary; circulatory; and other systems. The types of drugs were classified as sufentanil and non-sufentanil mainly considering the fact that sufentanil itself accounted for nearly 90% of the drugs in this study and we aimed to obtain more direct clinically meaningful findings. The results were expressed as means ± standard deviation for continuous variables or as number and percentages for categorical variables. Continuous variables were compared using Student’s t-test and analysis of variance (ANOVA), while categorical variables were compared using chi-square test. In order to assess the impact of demographics and clinical characteristics on postoperative pain levels, we first applied univariate logistic regression analysis to screen for demographic or clinical factors that might be associated with postoperative pain, and variables were selected based on statistical significance or possible clinical importance. Then, multinominal logistic regression models were developed to explore the various risk factors associated with pain level. All *p*-values were based on two-sided tests with statistical a significance level set at 0.05. The above analyses were performed using the SAS statistical package ver.9.4 (SAS Institute Inc., Cary, NC, USA).

### 2.5. Ethical Statement

Our research protocols were approved by the institutional review boards of the Affiliated Suzhou Science and Technology Town Hospital of Nanjing Medical University (IRB202106001OI). The implementation of the current study adhered to the tenets of the Declaration of Helsinki of the World Medical Association regarding scientific research on human subjects. Information was collected on a total of 11,383 patients who met the study criteria, and personal records of all participants were removed prior to analysis in order to maintain anonymity.

## 3. Results

During a 6-year study period, a total of 11,383 cancer patients who underwent surgery were included in our analysis. Of these patients, 5237 (46.0%) were male and 5628 (49.4%) were female, ranging in age from 18 to 96 years. About 22.9% (*n* = 2604) patients underwent endoscopic surgery. The largest proportion of surgical sites were in the digestive system (40.9%), followed by the reproductive system (21.6%). About 82.7% (*n* = 9409) patients were treated with sufentanil-based drugs. There are more details about the frequencies and proportions of each study variable depicted in Table 1.

Pain evaluations were respectively performed on patients at the 24th, 48th and 72nd hour after surgery and the number and proportions of each pain group are shown in Table 2. At the 24th hour after surgery, the overall pain incidence was 76.9% (76.1%, 77.7%), with mild pain being 61.8% (60.9%, 62.7%) and moderate to severe pain being 15.1% (14.5%, 15.8%). Over the three pain evaluations performed after surgery, the proportion of patients with no pain and mild pain increased, and the proportion of patients with moderate to severe pain decreased significantly. The specific changes in the proportions can be seen more visually in Figure S1 of the Supplementary Material.

Table 2 also shows the incidence of various adverse reactions in patients at the three postoperative evaluations. Overall, the incidence of adverse reactions was low, except for uroschesis (47.6–83.0%), which was a common postoperative symptom. The incidence of nausea and vomiting (1.7–6.1%) was slightly higher than that of excessive sedation (0.8–1.9%) and pruritus (0.1%). Although the incidence of some adverse reactions increased slightly at the 48th hour after surgery, the overall incidence of various adverse events tended to decrease over the three evaluations within 72 h after surgery.

Table 3 presents the relationships among the demographic characteristics and clinical factors of patients with different levels of pain. Multinominal logistic analysis showed that several variables were associated with mild/moderate to severe pain, including gender, age, surgical modality, system in which the surgical site was located, and type of medication used. Females were at higher risk for mild pain and moderate to severe pain as well, especially at the 24th hour after surgery (mild pain: OR 1.58, 95% CI 1.26–1.98, *p* < 0.001; moderate to severe pain: OR 1.37, 95% CI 1.07–1.75, *p* = 0.013). Age and weight were associated with only moderate to severe pain intensity (age: OR 0.98, 95% CI 0.97–0.99, *p* = 0.003; weight: OR 1.02, 95% CI 1.01–1.03, *p* = 0.034). At the 48th hour postoperatively, endoscopic surgery was observed to be associated with a higher risk of both mild pain (OR 1.40, 95% CI 1.13–1.74, *p* = 0.003) and moderate to severe pain (OR 1.56, 95% CI 1.18–2.05, *p* = 0.0016). In the three postoperative evaluations, patients with surgical sites in the respiratory system had a higher risk of pain when compared to the digestive system (mild pain: OR 1.35, 95% CI 1.09–1.68, *p* = 0.007; moderate to severe pain: OR 2.13, 95% CI 1.37–3.33, *p* < 0.001), and other patients had a relatively lower risk of pain. Lower risk was noted for both mild pain (OR 0.72, 95% CI 0.60–0.87, *p* < 0.001) and moderate to severe pain (OR 0.57, 95% CI 0.37–0.89, *p* = 0.013) at the 72nd hour postoperatively for patients treated with sufentanil.

## 4. Discussion

In this retrospective study, we investigated the incidence of postoperative pain and adverse reactions in a total of 11,383 patients who underwent surgery between 1 January 2014 and 31 December 2019 and further explored the associated risk factors. We found that at the 24th hour after surgery, the overall pain incidence was 76.9%, with the mild pain being 61.8% and moderate to severe pain being 15.1%. Postoperative pain, especially moderate to severe pain, undoubtedly causes great distress to patients, and the higher incidence of pain also suggests that we may not be providing adequate analgesic treatment to patients in postoperative pain management. The results of a meta-analysis of persistent pain after breast cancer surgery showed that when patients reported a 36% incidence of pain, clinicians assessed only a 23% incidence of pain [17]. Patients’ postoperative pain is severely underestimated. In addition, some studies have shown that, besides inadequate analgesia, anxiety states were also associated with an increase in postoperative pain, which can lead to a decrease in the patient’s pain threshold [18,19]. As for adverse reactions, at the 24th hour postoperatively, the incidence of uroschesis was the highest one (80.3%), followed by nausea and vomiting (6.1%), with others in a much lower incidence. At the 48th hour after surgery, they maintained a similar level, but decreased significantly at the 72nd hour after surgery. Factors such as being female, being young, undergoing endoscopic surgery, having the surgical site in the respiratory system, and using non-sufentanil drugs were associated with an increased risk of pain in patients with both mild intensity and moderate to severe intensity.

In our study, females had a higher risk of postoperative pain than males, which was particularly evident at the 24th and 48th hour after surgery and especially for mild pain. This finding was in line with existing results of other studies in which gender as female was a risk factor that increased the intensity of postoperative pain [20]. However, at the 72nd hour after surgery, there was no longer a statistically significant difference in pain levels between males and females. One possible reason could be that females may be more sensitive to pain after surgery and were more likely to report their pain to their health care provider after the onset of pain in order to obtain effective analgesic treatment. Thus, females who receive relatively more adequate analgesic treatment had a decreasing risk of pain, and at the 72nd hour postoperatively, there was no longer a significant difference in postoperative pain risk from males. A study of gender differences in postoperative pain in patients showed that females were more likely to report high levels of pain and had a higher risk of pain than males up to 48 h after surgery, but the difference in postoperative pain decreased over time and eventually reached the same level as males. This study suggested that although acute pain was greater, potential recovery-related or treatment-related factors may prevent more severe acute pain symptoms in females from worsening their long-term prognoses [21]. This conclusion may provide a reasonable explanation for the trends in pain-risk differences between males and females in our study.

Our study found that older adults had a lower risk of moderate to severe pain. Several studies have demonstrated that age was associated with postoperative pain [13,22], Being younger is associated with a higher risk of pain; with all else being equal, older adults have a lower risk of pain [13]. This phenomenon may arise because older adults have higher pain thresholds and more peripheral neuropathy, all factors that contribute to pain reduction [13]. In addition, older patients have an increased sensitivity to opioids, which would facilitate postoperative pain control and lead to a lower risk of pain in such patients [23,24]. It has been shown that older patients have reduced nociceptive function in their peripheral nerves, so in some cases they experience reduced pain symptoms and have a reduced need for opioids [25].

Our findings showed that endoscopic surgery was associated with a higher risk of pain. In the majority of these kinds of studies, as one would expect, patients undergoing endoscopic surgery had less intensity of postoperative pain compared to open surgery [26,27,28]. However, the statistics of our study showed that at the 48th hour after surgery, patients who underwent endoscopic surgery had a higher risk of pain than those who underwent non-endoscopic surgery. This result, although somewhat unexpected, is not an isolated case. There are relevant studies showing that some laparoscopic procedures are associated with high postoperative pain intensity [14]. Some studies have demonstrated that after many laparoscopic surgeries, patients often reported severe pain but did not receive any opioids at all or received opioids only in low doses, which supports the presumption that the painfulness of some laparoscopic interventions is underestimated [14]. Our findings suggested that the differences in pain levels and risks between the different surgical modalities were significant only at the 48th hour postoperatively. The possible reasons for this phenomenon are as follows. In both endoscopic and non-endoscopic surgery, anesthetic drugs were used during the procedure and the analgesic effect of the anesthetic drugs lasts for some time after the surgery; therefore, the difference in pain levels and risks between the two surgical modalities was not significant at the 24th hour postoperatively. Within 24–48 h postoperatively, patients who underwent endoscopic surgery were not given adequate analgesia because of insufficient attention to postoperative pain by health care providers, resulting in such patients having a higher risk of pain than other patients due to inadequate analgesia. During the 48–72 h postoperative period, on the one hand, patients undergoing endoscopic surgery who previously experienced pain due to inadequate analgesia may have fed back their feelings to the health care providers and subsequently received appropriate analgesic treatment; on the other hand, the less invasive surgical wounds gradually healed over time, so the difference in pain between patients undergoing different surgical modalities was not significant. 

We observed an association of pain intensity with surgical sites in the present study. Our results showed that the highest risk of pain occurred in patients with surgical sites in the respiratory system, followed by the digestive system, with a relatively low risk of pain in other systems. Related studies have shown that the type of surgery is also a risk factor associated with postoperative pain. The most painful surgeries were orthopedic major joint surgery, thoracic surgery, and open abdominal surgery [29,30,31]. This finding is generally consistent with the results of our study.

A wide variety of analgesic drugs had been administered to patients in our study, which were divided into two categories, sufentanil and non-sufentanil. In total, 9409 patients used sufentanil, which accounted for 82.7% of the total number of patients. The results showed that at the 72nd hour after surgery, patients using sufentanil had a lower risk of pain than those using non-sufentanil, however, there was no significant difference in pain level and risk between the two groups in the first two postoperative evaluations, which may be explained by the time required to accumulate the differences in the effects of the drug treatments.

We considered the large number of people in the study to be a strength of our research. In addition, compared to most studies that have focused only on pain within the first 24 h postoperatively, our study expanded the focus to 72 h after surgery. Therefore, a more comprehensive understanding of cancer patient’s postsurgical pain can be obtained, and the trend of changes in patient’s postsurgical pain level can be analyzed.

However, there are some limitations of our study. First, in this retrospective study, we were unable to obtain certain confounding factors regarding pain, such as psychological variables. It was also not possible to consider the variability of each case during the treatment process, such as the impact of different health care providers and different surgical incisions on pain. We ended up with a limited number of factors that could be included in the multifactor model for analysis. Second, the assessment of pain level was scored by patients based on their own pain perceptions under specific criteria, which may be biased to some extent due to inter-individual differences in pain sensitivity and tolerance. Third, at the 24th hour postoperatively, 2001 patients did not participate in the pain evaluation, accounting for 17.6% of the total study population, but the number of participants in the three pain scores was increasing. The reason for this phenomenon may be that some patients were experiencing very severe pain just after surgery and were unable to participate in pain scoring or did not have the will or energy to participate in scoring. This situation may cause the available statistics to be low compared to the true values. In other words, the incidence of pain and pain severity in patients may be underestimated. Fourth, postoperative pain thresholds vary by system and by type of surgery, and it may be interesting to perform a stratified analysis in this way. However, due to the overwhelming workload and reduced statistical power due to stratified analyses, a more detailed stratification analysis based on the system was not performed in the present study. Fifth, both static pain and dynamic pain were assessed postoperatively in our study, but we found that the incidence of static pain was low, and it was not significantly related to various risk factors. Therefore, we have only reported the dynamic pain distribution and associated risk factors. Sixth, our study did not address the specific dose of the drug used. Finally, the adverse reactions in our study included postoperative complications and drug side effects, both of which may cause nausea, vomiting, and uroschesis in clinical practice, so it was difficult to draw a clear line to distinguish them. 

## 5. Conclusions

In conclusion, postoperative pain still has a high incidence in cancer patients, and some patients may also experience very severe pain. The causes of postoperative pain in patients are very complex, and the results of this study showed that postoperative pain in patients was associated with factors, such as gender, surgical modalities, surgical site, type of medication and so on. High-risk groups for pain could be identified using these risk factors and targeted interventions could be made to reduce patients’ pain and improve their postoperative quality of life. 

## Figures and Tables

**Table 1:** Patients’ demographic data and surgical factors (N = 11,383).

Variables	*n*	%
Gender		
Male	5237	46.01
Female	5628	49.44
Missing	518	4.56
Age (years) (Missing = 518)	61.09 ± 11.33	
Weight (kg) (Missing = 570)	62.96 ± 11.48	
Operation modality		
Endoscope	2604	22.88
Non-endoscope	8261	72.57
Missing	518	4.55
System		
Digestive	4660	40.94
Reproductive	2461	21.62
Respiratory	1487	13.06
Other systems	1443	12.68
Urinary	537	4.72
Circulatory	230	2.02
Missing	565	5.00
Drug		
Sufentanil	9409	82.66
Non-sufentanil	1342	11.79
Missing	632	5.55

**Table 2:** Incidence of postoperative pain and adverse reactions in three evaluations.

Symptom	At the 24th Hour after Surgery	At the 48th Hour after Surgery	At the 72nd Hour after Surgery
Incidence of postoperative pain			
No pain ^†^	631 (5.54)	895 (7.86)	1694 (14.88)
Mild pain ^††^	7030 (61.76)	8188 (71.93)	8221 (72.22)
Moderate to severe pain ^†††^	1721 (15.12)	949 (8.34)	209 (1.84)
Missing	2001 (17.58)	1351 (11.87)	1259 (11.06)
Incidence of adverse reactions			
Excessive sedation	164 (1.44)	212 (1.86)	88 (0.77)
Nausea and vomiting	694 (6.10)	485 (4.26)	188 (1.65)
Pruritus	12 (0.11)	13 (0.11)	7 (0.06)
Uroschesis	9138 (80.28)	9445 (82.97)	5422 (47.63)
Missing	2003 (17.60)	1355 (11.90)	1261 (11.08)

Abbreviations: ^†^ NRS 0/10; ^††^ NRS 1–3/10; ^†††^ NRS ≥ 4/10.

**Table 3:** Multinominal logistic analysis of factors associated with pain in three pain evaluations.

	At the 24th Hour after Surgery		At the 48th Hour after Surgery		At the 72nd Hour after Surgery	
Factor	OR (95% CI)	*p* Value	OR (95% CI)	*p* Value	OR (95% CI)	*p* Value
No Pain ^†^ vs. Mild Pain ^††^						
Gender (ref: Male)						
Female	**1.58 (1.26–1.98)**	**<0.001**	**1.44 (1.19–1.74)**	**<0.001**	1.03 (0.90–1.18)	0.686
Age (per year)	1.00 (0.99–1.01)	0.715	0.99 (0.98–1.01)	0.063	1.00 (0.99–1.01)	0.867
Weight (per kg)	1.00 (0.99–1.01)	0.737	1.01 (0.99–1.02)	0.294	1.00 (0.99–1.01)	0.901
Operation modality (ref:Non-endoscope)						
Endoscope	1.21 (0.94–1.54)	0.133	**1.40 (1.13–1.74)**	**0.003**	0.92 (0.79–1.08)	0.320
System (ref: Digestive)						
Respiratory	1.27 (0.86–1.87)	0.229	1.26 (0.92–1.72)	0.144	**1.35 (1.09–1.68)**	**0.007**
Urinary	**0.33 (0.24–0.45)**	**<0.001**	**0.42 (0.32–0.55)**	**<0.001**	**0.42 (0.34–0.52)**	**<0.001**
Reproductive	**0.35 (0.27–0.46)**	**<0.001**	**0.46 (0.36–0.58)**	**<0.001**	**0.55 (0.46–0.65)**	**<0.001**
Circulatory	0.59 (0.33–1.06)	0.075	**0.61 (0.38–0.99)**	**0.045**	0.75 (0.51–1.09)	0.134
Other systems	**0.37 (0.28–0.48)**	**<0.001**	**0.52 (0.41–0.65)**	**<0.001**	**0.55 (0.46–0.65)**	**<0.001**
Drug (ref: Non-sufentanil)						
Sufentanil	1.02 (0.77–1.33)	0.913	1.10 (0.88–1.37)	0.397	**0.72 (0.60–0.87)**	**<0.001**
No pain ^†^ vs. Moderate to severe pain ^†††^						
Gender (ref: Male)						
Female	**1.37 (1.07–1.75)**	**0.013**	1.15 (0.91–1.46)	0.2443	0.98 (0.69–1.39)	0.906
Age (per year)	**0.98 (0.97–0.99)**	**0.003**	**0.98 (0.97–0.99)**	**0.0158**	0.99 (0.98–1.01)	0.208
Weight (per kg)	**1.02 (1.01–1.03)**	**0.034**	1.01 (0.99–1.02)	0.2182	1.01 (0.99–1.02)	0.237
Operation modality (ref:Non-endoscope)						
Endoscope	1.14 (0.86–1.50)	0.362	**1.56 (1.18–2.05)**	**0.0016**	0.83 (0.55–1.26)	0.388
System (ref: Digestive)						
Respiratory	**2.04 (1.36–3.05)**	**<0.001**	**1.78 (1.25–2.53)**	**0.001**	**2.13 (1.37–3.33)**	**<0.001**
Urinary	**0.12 (0.08–0.18)**	**<0.001**	**0.22 (0.14–0.35)**	**<0.001**	**0.25 (0.11–0.57)**	**0.001**
Reproductive	**0.14 (0.10–0.19)**	**<0.001**	**0.18 (0.13–0.25)**	**<0.001**	**0.11 (0.06–0.22)**	**<0.001**
Circulatory	**0.30 (0.15–0.60)**	**<0.001**	**0.28 (0.13–0.60)**	**0.001**	0.70 (0.27–1.88)	0.483
Other systems	**0.19 (0.14–0.26)**	**<0.001**	**0.24 (0.17–0.34)**	**<0.001**	**0.22 (0.12–0.43)**	**<0.001**
Drug (ref: Non-sufentanil)						
Sufentanil	1.00 (0.73–1.35)	0.974	1.04 (0.78–1.40)	0.770	**0.57 (0.37–0.89)**	**0.013**

Note: Multinominal logistic regression analysis of factors associated with mild and moderate to severe pain in three pain evaluations within 72 h postoperatively, with model containing six variables of gender, age, weight, surgical procedure, system in which the surgical site was located, and type of medication administered, with statistically significant results in bold. Abbreviations: OR—odds ratio; CI—confidence interval. ^†^—NRS 0/10; ^††^—NRS 1–3/10; ^†††^—NRS ≥ 4/10.

## Data Availability

The data that support the findings of this study are available from the corresponding author upon reasonable request.

## Supplementary Materials

The following supporting information can be downloaded at: https://www.mdpi.com/article/10.3390/curroncol30010065/s1. Figure S1. Incidence of postoperative pain in three pain evaluations.
