# Supplementary material for: Pain Incidence and Associated Risk Factors among Cancer Patients within 72 Hours after Surgery: A Large Retrospective Analysis
Source: Curr Oncol. 2023 Jan 8;30(1):854–64. doi: 10.3390/curroncol30010065 (PMC9858544; doi:10.3390/curroncol30010065)
Supplement: Supplementary file 1 [file curroncol-30-00065-s001.zip › curroncol-2063279-supplementary.pdf]

Current Oncology

Supplementary materials

Pain Incidence and Associated Risk Factors among Cancer Patients within 72 Hours after Surgery: A Large Retrospective Analysis

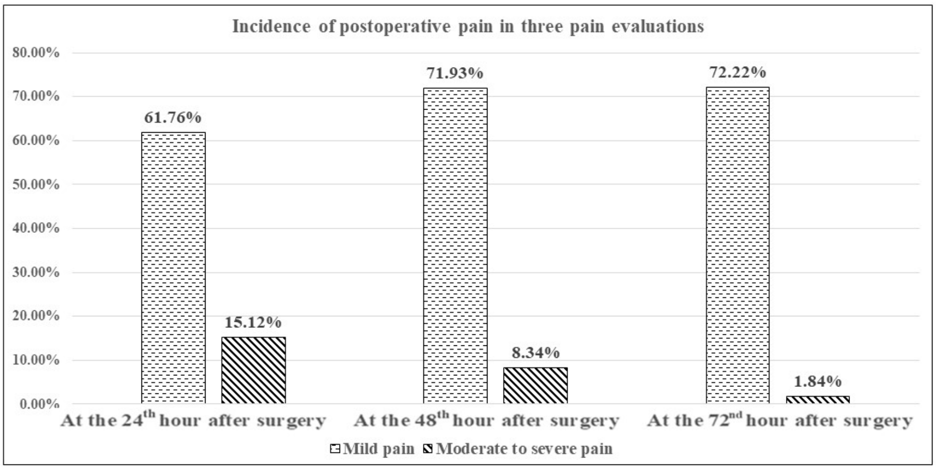

Figure S1. Incidence of postoperative pain in three pain evaluations.
